# Social work practice in complex emergencies: A study of Northwest Syria

**DOI:** 10.1177/14680173241283389

**Published:** 2024-10-15

**Authors:** Karen Paul, Myriam Denov, Lana Al Houssami

**Affiliations:** McGill University, Montreal, Canada; Independent Researcher

**Keywords:** Social work, social workers, community work, conflict, international social work, culture

## Abstract

**Summary:**

Existing scholarship discusses social work during war and conflict, but less social work scholarship specifically names and focuses on the reality of complex emergencies. This article discusses perspectives on “good” social work practice in complex emergencies by drawing upon a study of Northwest Syria which included 29 interviews with international and Syrian practitioners.

**Findings:**

This study found that although Syria did not have a formal social work profession before the emergency, the crisis created a need for social work practice to bolster and help reactivate Syrian families and communities’ supportive nature. Participants perceived “good” social work practices as a range of practices, that are suitable to the community, which helped to reactivate the family and community's supportive role. Practitioners described how “good” social work practices incorporated Syrian culture and were best performed by practitioners with certain characteristics. Ultimately, this article argues social work practice should aim to reactivate rather than replace these important and existing culturally embedded forms of support.

**Applications:**

During complex emergencies, “good” social work practice considers and incorporates cultural aspects such as spirituality and stigma. A range of social work practices, which are suitable to the community, can help to restore family and community supports. Practitioners who know the community, are trustworthy, and have a relevant background can best perform what are regarded as “good” social work practices.

## Background

Complex emergencies are characterized by extensive societal and economic destruction, massive displacement (Office for Coordination of Humanitarian Affairs [OCHA], 1999), weakened institutions (World Health Organization [WHO], 2024), mass causalities, loss of life, barriers to effectively responding, and high levels of danger toward humanitarian responders, all of which create needs that require unique forms of external support (Inter-Agency Standing Committee [IASC], 1994). The term “complex humanitarian emergency” was developed to define situations where multiple factors, including political and historical factors, make a setting particularly vulnerable to crises and influence the outcomes (Leaning, Briggs & Chen, 1999 as cited in Ventevogel et al., 2011). Keen (2008) highlights how the term “complex emergencies” draws attention to their unique conditions: “more generally, the term ‘civil war’ can prejudice our understanding of the complex fault-lines of conflict and the complex manipulation of violence for a wide variety of purposes, whereas the label ‘complex emergency’ draws attention to complexity and embodies a useful degree of vagueness about the *nature* of a violent conflict” (p. 2). The IASC, which is responsible for coordinating inter-agency humanitarian assistance (IASC, n.d.), defines a complex emergency as:

a humanitarian crisis which occurs in a country, region, or society where there is a total or considerable breakdown of authority resulting from civil conflict and/or foreign aggression; b) a humanitarian crisis which requires an international response which goes beyond the mandate or capacity of any single agency; c) a humanitarian crisis where the IASC assesses that it requires intensive and extensive political and management coordination. (IASC, 1994, p. 2)

Recent examples of complex emergencies include Ukraine, Yemen, and Northwest Syria (WHO, 2022).

Yet, the very nature, convolution, and intricacies of complex emergencies make it difficult to provide necessary support (IASC, 1994) and demand social work practice that is uniquely tailored to the context. While local social workers play a critical role in responding to the needs arising from the complex emergency (Bragin & Garcia, 2009), its impact on the family, community, and societal structures profoundly shapes the nature and direction of social work of practice. However, social work research has yet to document practitioners’ perspectives on what *they deem* to be effective social work practices in complex emergencies. To address this gap, by drawing upon the perspectives of practitioners working in the insecure context of Northwest Syria (NWS), this study explores perspectives on what is considered “good” social work practice in complex emergencies. Based on practitioners’ perspectives, this article asserts that social work practice can play a key role in helping to *reactivate* rather than *replace* the natural support systems that comprise the social fabric of families and communities in complex emergencies. To reactivate is “to make active or operative again; to restore to a state of activity, bring back into action” (*Oxford English Dictionary Online*, 2022, para 2). The IASC Guidelines for Mental Health and Psychosocial Support (MHPSS) in Emergency Settings acknowledge how communities impacted by emergencies often contain both informal and formal mechanisms which communities mobilize to address their needs. If these mechanisms are strained, *reactivating* these existing supportive structures is part of promoting a useful emergency response (IASC, 2007). As such, this article argues social work practices should not emerge to continually “take over” and replace the role of existing formal and informal structures, but instead, practitioners who perform social work functions can help to reactivate and revitalize rather than permanently take on culturally embedded, supportive family and community roles.

### The complex emergency in NWS

In NWS, since 2011, the ongoing complex emergency continues to profoundly impact Syrians through massive displacement, loss of life, and barriers to responding to the emergency. The United Nations Office for the Coordination of Humanitarian Affairs (OCHA, 2022b) documents that 2.8 million people are internally displaced in NWS. Furthermore, the Under-Secretary-General for Humanitarian Affairs and United Nations (UN) Emergency Relief Coordinator, Martin Griffiths, explained:

March marks 11 years of war in Syria, [and] said such devastation “finds few parallels” in recent history. More than 350,000 people have been killed, nearly 14 million have been displaced and basic services are destroyed. Meanwhile, civilians continue to be killed and injured along front-line areas in the country's north-west. (UN, 2022a, para. 4)

Exemplifying the barriers to effective response, Martin Griffiths also stated that not renewing the UN Security Council's authorization to provide cross-border assistance “will disrupt life-saving aid for the people living in north-west Syria” (UN, 2022b, para 17). The mass displacement, loss of life, and barriers to effectively responding create a complex situation in NWS.

Other characteristics of the complex emergency, such as strained institutions, economic and societal destruction influence social work practice in NWS. For example, the Humanitarian Needs Overview (2022) reports how shelling damaged health and educational facilities. OCHA (2022a) reports 8% of healthcare facilities have been destroyed, and only 422 of 624 health care facilities are functioning. Similarly, strained educational systems influence social work practice by creating new needs, such as a lack of education. OCHA (2022a) found that 44% of children in NWS were not in school. Moreover, not attending school has left children at risk of child labor and child marriage. Demonstrating the economic and societal destruction, OCHA (2022c) reports increased suicide due to financial hardship, domestic violence, or lost property. The strained institutions, societal and economic destruction represent profound challenges to engaging in supportive social work practice in NWS. In this context, this research explored what practitioners working within the complexity of NWS viewed as “good” social work practices.

### Social work in NWS

Although Syria did not have formal social work education prior to this crisis, international organizations introduced social work roles to support displaced Syrians. For example, to address child protection, gender-based violence, and MHPSS concerns, existing programs now incorporate social work functions such as case management (Syria Protection Cluster (Turkey), 2019). Similarly, organizations developed the psychosocial worker (PSW) role to address the gap in psychosocial support services (Nemiro et al., 2021). In addition, an estimated 280 psychosocial workers provide MHPSS services in NWS (World Health Organization Regional Office for the Eastern Mediterranean, n.d.) and some Syrian PSWs have received psychological guidance counseling degrees from Syrian universities. Having Syrians perform social work functions in NWS, without formalized social work education allows for a unique exploration of how effective social work practice can emerge during crises.

### Social work research in complex emergencies

Although there is a growing body of literature addressing social work in conflict settings (Bragin et al., 2016; Semigina & Gusak, 2015), there is only one known social work study in English that specifically focuses on complex emergencies (Bragin & Garcia, 2009). The available research on social work practice in complex emergencies may be less apparent for a few reasons. First, conducting research in such settings is challenging because accessing communities within active war zones is difficult and dangerous, making empirical research desperately needed, yet often unattainable. Second, social work research has more frequently addressed the realities of political conflict (Sonnenberg & Ghaderi, 2021), armed conflict (Bragin et al., 2016), and war (Maglajlić et al., 2008). Thus, there is a need for social work research to ensure the inclusion of the perspectives of practitioners with experience in complex emergencies to inform social work practice.

Although social work research on complex emergencies has underscored the relevance of the social work profession in such crises (Bragin & Garcia, 2009), the need to pay particular attention to the unique culture and context has been emphasized (Bragin et al., 2016; Harrop & Ioakimidis, 2018). Based on a study comparing the competencies required by child protection organizations working in complex emergencies with social work competencies, Bragin and Garcia (2009) recommend that social work should increase its role in strengthening local capacity to promote culturally relevant, effective practice with youth impacted by complex emergencies. Similarly, research on social workers in the West Bank and East Jerusalem stated the social work education is mainly based on Western social work theories, yet Palestinian social workers do not always follow these Western theories in practice (Harrop & Ioakimidis, 2018). Both studies highlight the need for greater attention to culture and context as well as the necessity to explore what local practitioners believe to be “good” social work practices in complex emergencies.

In response, this research explores practitioners’ perspectives on “good” social work practice, and participants’ explanations as to why they represent a “good” practice. The Food and Agricultural Organization of the UN (Food and Agriculture Organization of the United Nations [FAO], 2016) emphasize that “good practices” are ones which produce good results and can be thereby recommended as models. However, determining what is considered “good practice” is inherently variable, and deeply impacted by local culture, history, economics, geography, power relations, and must be contextualized to the broader socio-historical context. What is considered “good” in one context, may not be in another. Moreover, using the terminology of “good” practice may be problematic, as it has sometimes been used by social workers to justify abusive practices (Ives et al., 2020). This highlights the importance of considering whose voices inform social work and the need for practitioners’ perspectives from diverse contexts to carefully strengthen social work practice.

## Method

Ethical approval for this study was received from the McGill University Ethics Board (REB File No. 42-0619). The well-being and anonymity of participants were assured via multiple strategies. A security consultant provided information on secure communication methods. To ensure anonymity, participants’ demographic information was not collected. Furthermore, participants provided verbal consent for interviews and could choose to participate with their name or a pseudonym; and to be audio recorded. Two Syrian peer researchers (PRs) conducted interviews in Arabic to ensure culturally appropriate support. Although the interviews were designed to not ask about distressing topics, sometimes difficult topics arose during the interviews because of the challenging environment. In response, having Syrian PRs was important. For example, when discussing a challenging topic during an interview, a participant told the Syrian PR: “when I talk, you know how it is.” Nonetheless, if participants experienced distress from the interviews, clinicians were identified who could provide additional support in Arabic or English.

The research included four phases from 2020 to 2022: (1) Developing a Community Advisory Board (CAB), (2) A Pilot Study, (3) Interviews, and (4) Data analysis.

1. *Developing a CAB:* This study was guided by collaborative community-based research methods (Israel et al., 1998) in an online format. Community-based research methods recognize the importance of involving a range of actors from the community in the research (Israel et al., 1998) which supports the use of a CAB. The CAB ensured that culture, sensitivity, and ethics were an integral and ongoing part of the project by providing their input on the research through a pilot study. Six Syrian and seven international practitioners familiar with NWS joined the online CAB. International staff/practitioners can be defined as “all staff not from the country within which they are working,” and may include staff from nearby countries and who have experience working as national staff in their own country (Stoddard et al., 2011, p. 3).

The CAB population included practitioners such as community health workers, case managers, PSWs, social workers, managers, and technical advisors with remote or in person experience related to a specific area of NWS. The research occurred in a specific area of NWS, which is notable as the needs might be different according to the context. The specific area is available upon request due to the sensitivity of the situation and the area as well as to protect participants’ safety. Practitioners working in gender-based violence, health, rehabilitation, MHPSS, protection, and child protection were all included.

CAB participants were recruited using convenience sampling techniques (Palinkas et al., 2015) by sending an invitation to eligible participants by drawing upon existing networks and purposeful sampling techniques (Palinkas et al., 2015) by sending invitations to relevant coordination group leaders.

2. *Pilot study:* The pilot study invited CAB members to participate in an interview and provide feedback through an online survey. Seven CAB members (four international practitioners; three Syrian practitioners) participated in semi-structured interviews in 2020. Participants received FAO's (2016) definition of a “good” practice and the interviewer reviewed the aspects of the social work's definition and core purposes. The interviews explored participants’ perspectives on what constitutes “good” social work practices with individuals, families, groups, and communities; why these practices were considered as “good”; how to effectively support Syrian practitioners; and recommendations for “good” social work practices in other complex emergencies. The interviews lasted from 30 to 120 min and were conducted via Skype, WhatsApp, Wire, or Telegram. Ten CAB members completed the survey. Based on the pilot study feedback, the research added an interview question asking how participants would describe social work in NWS, and provided participants the choice to interview with a male or female Syrian PR.
3. *Interviews:* In addition to the population previously described for the CAB, the population for these interviews also included community members with experience providing or receiving care related to the core purposes of social work (Sewpaul & Jones, 2004). These participants were identified using convenience, purposeful, and snowball sampling techniques (Palinkas et al., 2015), by sending invitations through professional networks and relevant coordination group leaders. Twenty-two participants (two international practitioners; 20 Syrian practitioners) completed interviews in 2021. The third author completed 19 interviews in Arabic and the first author conducted three interviews in English.
4. *Data analysis:* The first author analyzed the data using constructivist grounded theory (CGT) (Charmaz, 2014). CGT is a contemporary form of grounded theory which focuses on research relationships and subjectivity, gaining “abstract understanding of studied life” (Charmaz, 2014, p. 342). CGT was accomplished through initial and focused coding, writing memos, establishing categories, and creating diagrams to explain the emerging theory (Charmaz, 2014). To ensure rigor, the third author also wrote memos and the first author discussed the analysis with the Syrian PRs. To conduct initial coding, each transcript was read and a memo was written describing the interviews’ meaning; and then coded line by line to focus on the participants’ meanings and actions (Charmaz, 2014). Next, data were coded segment-by-segment, allowing for more focused codes to emerge, and focused codes were then grouped to develop categories and revise the diagrams.

## Findings

**Figure 1.**
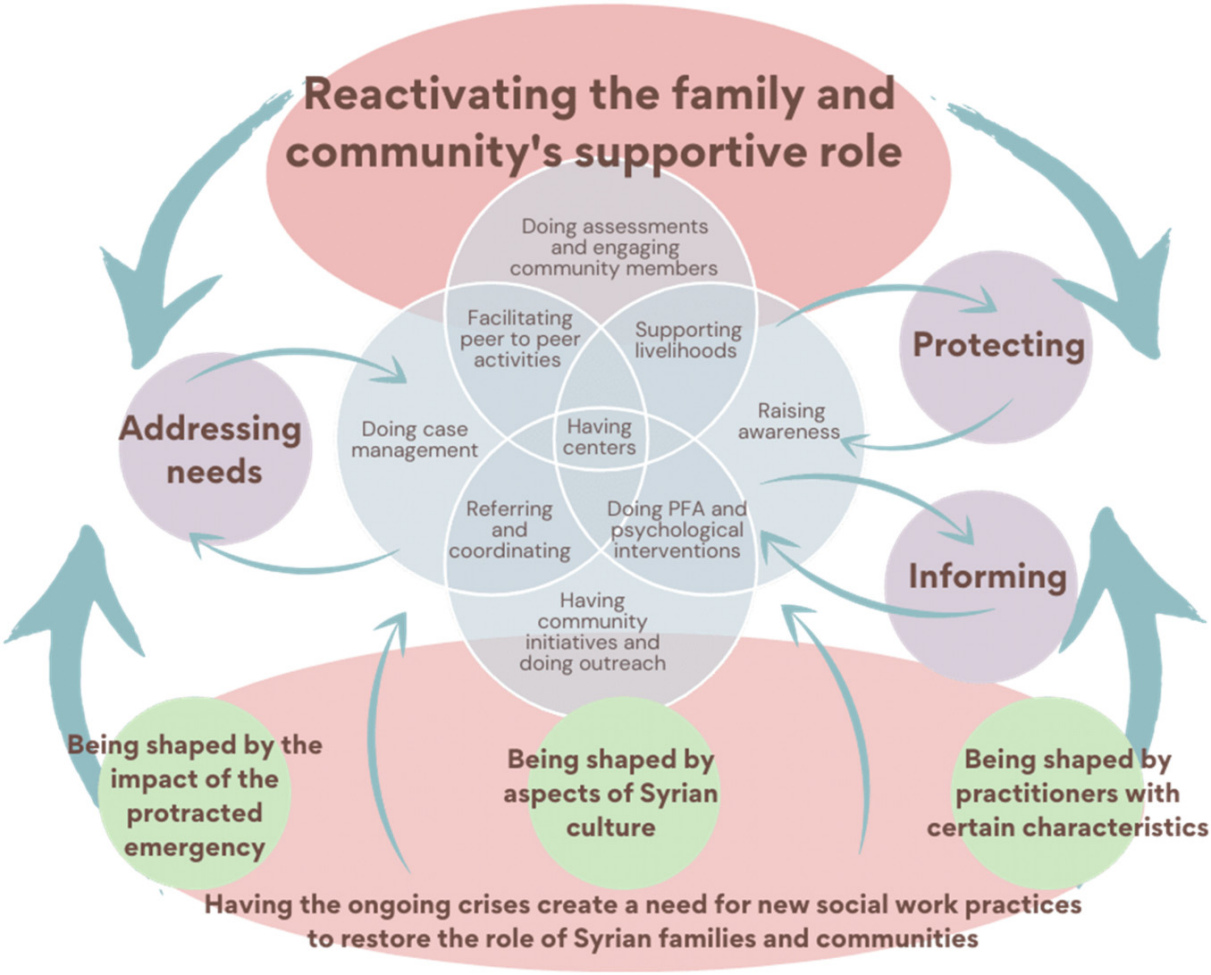
Perspectives on good social work practices in Northwest Syria.

This article reveals three key findings which suggest that social work, as an emerging profession, can help to *reactivate* rather than *replace* the socio-historical and cultural support systems that comprise the social fabric of families and communities in society during complex emergencies as illustrated in Figure 1. First, Syria did not have a social work profession prior to the start of the complex emergency. However, the ongoing crises of war and displacement created a need for social work practices to emerge and to help restore the critical and supportive role of Syrian families and communities. Second, practitioners considered “good” social work practices as those that helped to reactivate the family and community's supportive role through a range of practices including peer group activities, livelihood support, case management, awareness raising, community initiatives, and centers such as spaces for women, youth, vocational training, and psychosocial support. Practitioners highlighted key actions of “good” social work practices as reactivating the family and community as well as informing, protecting, and addressing their needs. Practitioners also perceived “good” social work practices as providing an overlapping impact from the individual to the family and community. Third, effective social work practices were considered those that reflected and embedded Syrian culture and included local understandings of spirituality and stigma. Practitioners suggested that effective practices were those that are best performed by practitioners who know the community, are trustworthy, and have related experience and education. The study's findings ultimately suggest social work matters in complex emergencies and can even be lifesaving. Yet, it requires careful consideration of who is conducting the practice and how the practices are aligned with the culture and context, particularly in NWS that did not previously have a formal social work profession.

### Complex emergencies and the need for new social work practices

Practitioners described the integral and supportive role of family and communities in Syrian culture:

The family has a big role in the social support process…. We rely a lot on the family and friends role because they have a big role within the local community. (Syrian Practitioner C)

Practitioners also explained the deleterious impact of the crisis on these supportive family and community networks, creating a vital need for new forms of support:

Because of the shattered community … there is now a conspicuous need for social work, now people started to appreciate, to understand the value of this sort of work. (Syrian Practitioner A)

Despite the impact of the crisis, a Syrian practitioner highlighted how the family continues to play an important role:

The family has a huge role in enhancing the recovery and psychological wellbeing and offering self-care … after nine years of … crisis, the family is no longer able to offer enough care and in a good way…. However, the family and parents still have a huge role. (Syrian Practitioner C)

Furthermore, practitioners explained how social work was regarded a new phenomenon:

At the beginning of the war was when the organizations work began between 2012 and 2013 … people were not accustomed and even organizations were not accustomed to a disaster of such magnitude … there was no clear concept of social work. (Syrian Practitioner D)

Ultimately, participants explained how the family and community play an integral and supportive role in Syrian culture. Although the emergency strained these critical networks, these roles were not completely diminished, creating a vital need for new social work practices to recognize and build on culturally embedded supportive roles.

### Practitioner perspectives on “good practice”

Practitioners suggested that “good” social work practices were those that helped to reactivate family and community contributions, through a range of practices such as peer group activities, centers and livelihood support, case management, awareness raising, and community initiatives. Since Syrian families and communities supported each other prior to the crisis and social work practices are relatively new, practitioners emphasized how “good” social work practices re-enabled families and communities to continually address their own needs. These “good” practices worked to revitalize the family and community's supportive role by reactivating economic and social life, the individual's role in the family and community and the community's role.

#### Peer group activities

Practitioners described peer group activities—such as—a process of gathering together those who share the same age, gender and were experiencing similar problems. This is accomplished to exchange experiences, information and engage in collective activities, facilitating the development of ongoing relationships and support. *Syrian Practitioner D* described different types of peer group activities: “we do men's soccer for example a cooking group for women anything that comes to your mind with creativity.” The difficulties with humanitarian access, lack of human resources and the overwhelming, diverse needs in NWS make facilitating ongoing support critical:

You are also bringing clients and families together that you would otherwise be seeing separately and you are trying to create a mutual social support network between them and a peer network that can exist and can be supportive and mutually reinforcing as a network without the social worker being present. And that is particularly key in Northwest Syria because of the humanitarian access issues. (International Practitioner B)

*Syrian Practitioner H* shared that “through the group activity we are strengthening the feeling of belonging, encouraging sharing and exchanging ideas, enhance social communication, strengthen individuals’ self-esteem,” illustrating how initiating peer group activities helped restore Syrian's social life and roles in the community.

#### Centers and livelihood support

Practitioners described the ways in which centers, which included spaces for women, youth, vocational training, and psychosocial support, can help to revitalize Syrian's social and economic life as well as their roles in the family and community. Considering the impact of the crisis on societal structures, centers were also said to contribute to restoring Syrians’ social life:

When they used to go and visit one of the community centres, empowerment centres … they used to feel like “we are back to something beautiful in life, we are gathering again outside the camp and going out.” (Syrian Practitioner I)

Given the profound economic destruction, centers provided spaces for rehabilitating Syrian's livelihoods, re-enabling Syrians to address their needs:

We saw success stories and success cases in the economy recovery projects … I heard it a lot … my materialistic status was over the wind [excellent]…. “I was a sultan but now humiliation, oppression and need … people's help”. So really when these services are provided in a professional and correct manner, in a way that guarantees beneficiaries access to it. So … he would tell you that indeed today I have changed, I became a new person, I can actually do something to my community, I am not a burden, I don’t need anyone. (Syrian Practitioner K)

Furthermore, another practitioner described why having vocational centers for youth and other practices to support families restores the community's role:

All these practices are contributing to enhance the well-being and increase the capacities of the community to rise on its own more effectively. (Syrian Practitioner H)

#### Case management

Practitioners expressed the ways in which case management, which can be provided in centers, had the capacity to revitalize the individual's role in the family and the community, reflecting how practitioners perceived “good” social work practices as providing and ensuring an overlapping and intersecting impact from the individual to the community:

So we as PSWs we help with these issues and put a plan for the individual, a treatment plan. We will put it with the beneficiary, until they reach the level of recovery. *They can go back to be an active individual in their community* [emphasis added]…. These practices and these activities helps them to deal with these pressures and problems. (Syrian Practitioner H)

One Syrian practitioner highlighted how case management could be lifesaving for those who were at imminent risk of suicide and help people return to their role in the community:

We consider [case management] like lifesaving activities during suicide attempts or even on the level of her psychological status it became better…. Those people could be integrated, she could be a girl so she would be integrated or returned back to school or education. (Syrian Practitioner K)

#### Awareness raising

Practitioners also described raising awareness as a “good” social work practice that encourages the individual's role in the family and community and reflects the key action of informing. Considering that the crisis impacted families and created new problems and challenges, one practitioner described how awareness raising contributed to restoring a sense of coherence in families:

The most important thing for the families and if you focus on it is awareness raising sessions. You can’t imagine what awareness raising sessions could do…. Because they are introducing a community—let's be honest before these things they didn’t have much of a big issues…. Awareness raising sessions for the families are necessary and very important and it also includes how we teach them parenting skills…. We are trying to help them understand [that] the more you come together as a big family, the more you would be together and you can help and support each other. (Syrian Practitioner D)

Furthermore, this same practitioner highlighted how the practitioners’ role is to increase the connection between families and communities during awareness-raising sessions:

When the person supervising the group nourish this sense of humanity in them and you are a big family the situation would become easier, and … the more recluse the family gets and they face their problems by themselves then the situation would get much more difficult, so our job is to remove the obstacles between the families and remove the obstacles between the displaced and residents or the hosting village or community, the more they get closed to each other the better the results would be. (Syrian Practitioner D)

#### Community-led initiatives

Practitioners shared how the community should conduct social work with or without the presence of organizations, indicating the importance of new social work practices emerging to reactivate rather than replace culturally embedded family and community supportive mechanisms:

We through training, raising awareness, coordination, and organizing within the community we can achieve social work regardless of the presence of organizations or not…. We should as a community work on the concept of social work as a whole, what risks it involves? What difficulties and challenges does it have? And through social work we should solve things in this community and don’t be dependent on organizations and other people. (Syrian Practitioner F)

Practitioners shared how community-led initiatives helped to restore the community's supportive role:

What makes them a good social work practice is that they gave back the responsibility of protection of children to the community…. The community level did not do enough and they were not empowered enough to take control to take care of their own to shoulder the responsibilities they should be. They were not even aware that these were their responsibilities. So, the NGOs did this community-based intervention where they trained some individuals from the community some local councils some etc and … they enabled like empowered them … to shoulder the responsibility that they should be undertaking. (Syrian Practitioner A)

In another example, a practitioner described a community initiative to support education:

These initiatives are genuinely saving many cases. Like the last initiative I did [with] one of schools in the community. Their financial support was cut from the beginning of the school year. The teachers didn’t receive it any salaries at all. So, we did an initiative which is collecting money from parents of students after taking the consent from the Minister of Education…. Many schools have emulated in order to secure teachers’ salaries so that the educational process can continue and so that the children or students don’t face or don’t be end up on the streets and girls are not exposed to early marriage and violence and cases of exploitation. (Syrian Practitioner O)

One practitioner explained a community initiative called *kafala*, which can be similar to kinship care, to support children to stay with their families. *Kafala* is a child protection measure, prevalent in Islamic legal countries, which can be formal and involve a competent body or informal in nature. *Kafala* occurs when a person, who is called the *kafil*, voluntarily cares for the specific needs of a child deprived of their family. The practitioner described facilitating Kafala because an orphanage had closed:

We tried to recommunicate with the parents, for example if there's Kafala for this child then the mother can take care of this child. There was a large number of children who we were able to return them to their mothers … after coordination with several organizations and many people received a lot of support for those orphan children. (Syrian Practitioner L)

### The importance of Syrian culture and local Syrian practitioners

Practitioners considered “good practices” to be rooted in Syrian cultures. For example, practitioners emphasized the need to consider religion and spirituality:

When you want to do good social work practices, the first thing is … something matching and suitable to the community you are working in … if you come through religion, morals, customs and traditions, they would accept you more here in our community. (Syrian Practitioner G)

Alongside the importance of religion, practitioners described the need to consider the stigma associated with seeing a social worker. A practitioner explained how offering social work within centers appeared to carry less stigma:

These physical spaces enable social workers to operate … to neutrally meet a client where they are not going to be stigmatized. (International Practitioner B)

Participants shared how practitioners should have direct knowledge of the community:

One should have a good comprehension … to the culture in the community … For example one of the things that could happen—the community could consider the person is possessed but he's not mentally ill, it's easier for him to be considered possessed. This person is possessed but it's impossible that he's mentally ill. So the social worker should understand this situation and know that this the culture in the community to be able to deal with people … explain the situation in a way that is suitable to his culture. (Syrian Practitioner Q)

According to practitioners, being trustworthy to clients and communities is the core of their ability to perform effective social work practice:

Our community is known for its customs and traditions, most people know each other, I mean people … the stigma matter is really important for us and especially for the people who’ve been subjected to violence. We are in war and the word war means that you expect all types of violence could happen and all types of difficulties, challenges, and psychological pressures—so what makes them good is because there's confidentiality, there's respect, there's trust: building trust between the service provider and beneficiary. (Syrian Practitioner O)

Participants highlighted the importance of having relevant education, experience, training and capacity building:

These people we can say who worked in 2011 their experiences are not like now. I mean now we’re in 2020, I mean nine years gave them long-established experience. There are training courses and certain programs available to acquire skills and experiences and capacity building and this will affect the community. (Syrian Practitioner C)

The data illustrated the ways in which practitioners believed that performing “good” social work practices were conducted by those who know the community, are trustworthy, and have relevant experience and education contribute to promoting effective social work practices.

## Discussion

This study explored practitioners’ perspectives on what are considered “good” social work practice in complex emergencies through a study of NWS. Local social workers and paraprofessionals who perform social work functions play a critical role in responding to the needs of families and communities affected by complex emergencies (Bragin & Garcia, 2009). The findings highlight the sophistication of Syrian practitioners’ ability to perform social work practices which have been locally established over time and validated by experience. This study reflects how practitioners rooted their knowledge of “good” social work practices within how Syrian families and communities can support themselves. In complex emergencies such as NWS, the long duration of the crisis, strained institutions, limited external aid and mass displacement makes reactivating the family and community's supportive role even more critical because there are few alternatives. The crisis strained the family and community's supportive roles, yet these roles were not completely diminished and maintained their importance within Syrian culture, creating an essential resource for social work practice to build upon. Although the Syrian family and community's integral role may not return to the way it was before the crisis, social work practice can help to build upon existing Syrian forms of support rather than emerging as a practice that fully takes on these roles. This is critical as building upon available resources, including remaining existing systems, supports a more culturally relevant and sustainable response (IASC, 2007).

Reactivating the family and community's supportive role is consistent with the IASC Guidelines (IASC, 2007), and providing multi-layered MHPSS which is depicted through an intervention pyramid. The second layer of this pyramid describes how supporting a portion of the population to access critical community and family supports can promote affected persons to sustain their mental health and psychosocial well-being (IASC, 2007). These guidelines acknowledge how an emergency disrupts family and community systems, and that affected persons will benefit from support to access any remaining family and community supports (IASC, 2007). The IASC Guidelines (2007) highlight the following helpful interventions to strengthen family and community supports: supporting livelihoods, activating social networks through groups for youth or women, and educational activities which align with the practices suggested as useful in this study. Furthermore, the IASC Guidelines (2007) emphasize the importance of community mobilization and taking steps “to activate and strengthen local supports and to encourage a spirit of community self-help” (p. 100). In keeping with these guidelines (IASC, 2007), this study's findings illustrated how social work practice can contribute to restoring culturally embedded family and community supports. Similarly, Ventevogel and Whitney (2023) argue that “in the blossoming field of humanitarian MHPSS, social workers have a unique and critical role to play in keeping the heritage of community empowerment alive” (p. 15). Bragin (2020) acknowledges how in emergency settings, social workers who are not from the affected context should work to reactivate existing systems of support which includes local practitioners. This research offers additional evidence highlighting the importance and ability of social work practice to effectively help to restore families and communities’ supportive mechanisms during crises.

Social work scholarship highlights how families and communities fulfilled social work functions prior to the emergence of social work in Afghanistan (Bragin et al., 2016), Iraq (Faraj, 2021), and Sri Lanka (Herath, 2017). In Afghanistan, Afghan traditions include the role of extended families and communities to provide protection for children, and community leaders offer additional support as required (Bragin et al., 2016). In Sri Lanka, community support is historically embedded in Sri Lankan culture, and a culture of community care was present before professional social work began (Herath, 2017). Similarly, in Iraqi-Kurdistan, the roots of social work and caring for others are central to Kurdish culture, but the social work profession started in 2007 (Faraj, 2021). In Iraq, the war's impact on society contributed to the need for social work (Faraj, 2021). Expanding on this knowledge, this current study offers additional insights into how social work practice could emerge to reactivate rather than replace traditional family and community support mechanisms.

Furthermore, this study highlighted how practitioners’ in-depth knowledge of the relevant cultures and communities fostered effective practice in NWS, which builds on concepts in existing social work literature. Social work literature describing social work practice during a time of conflict in Bosnia and Herzegovina (BiH) (Maglajlić et al., 2008) also reflects the importance of practitioners who know the context and culture. In BiH, the international agencies developed a parallel social welfare system, created mainly by locals who could speak English instead of local social workers. Existing scholarship also emphasizes the importance of incorporating aspects of culture such as spirituality into social work practice in Palestine (Lindsay, 2007) and Afghanistan (Bragin et al., 2016). This study demonstrates how a range of locally based social work practices, rooted within the supportive elements of cultures, societal structures, spirituality, and beliefs across time can help to reactivate the family and community's supportive roles.

This research highlights tensions described in existing social work literature about the nature of social work during crises. Lavalette (2011) questions what constitutes professional social work and whether people need formal training as professional social workers to perform social work practice, questioning who should be considered social workers. Lavalette (2011) describes how during crisis situations a form of social work practice called “popular social work” emerges. Crisis situations create an urgent need to establish forms of social work practice that help communities to address their needs (Lavalette, 2011). Thus, the findings of this research study also raise questions about what is considered “social work” in NWS and who can be considered “social workers,” particularly in the absence of formal social work education. The findings suggest that a fundamental characteristic of social work is not to take on the functions of families and communities, but to support families and communities to resume these functions themselves.

This study also highlights that while popular social work should inform effective social work practice, not all popular social work may be useful for communities. For example, Ukrainians developed volunteer organizations to address the basic needs of internally displaced persons. However, some of these volunteer organizations needed more systematic approaches, qualified service providers and more empowering strategies to help address the needs of internally displaced persons (Semigina & Gusak, 2015). Furthermore, research in other conflict settings has noted how family can be both a source of support and a potential harm (Akesson & Basso, 2022). Global guidelines also recommend the use of local practices to offer support, but also caution to not “assume that all local cultural practices are helpful” (IASC, 2007, p. 15). Although popular social work's effectiveness should be carefully considered, it can still add value. Providing an excellent example of this process is the work to develop a social work curriculum in Afghanistan based on the existing role of Afghans who performed social work functions (Bragin et al., 2016). As such, research and practice must continue to identify the supportive aspects of families and communities for social work practice to build upon in order to successfully empower individuals and communities to jointly address their own needs.

This study offers insights for other complex emergencies on how social work practice can contribute to restoring the social fabric. Existing scholarship prioritizes promoting “community-based psychosocial methods that focus on social connectedness and interpersonal ‘healing’” in humanitarian contexts (Jones & Ventevogel, 2021, p. 3). Yet, a research gap is how to successfully strengthen local community-level social supports (Tol et al., 2020). This study offers insights into how social work practice may help to restore this social fabric in complex emergencies by supporting the revitalization of the family and community's supportive role through a range of practices that are suitable to the community, including a focus on centers, livelihoods, case management, raising awareness and peer group activities.

## Limitations of the study

For reasons of security and confidentiality, this research did not specifically ask participants to identify as a beneficiary, community member, or practitioner or collect participants’ demographic information. As such, this study has limited knowledge of how representative the sample is of different organizations, genders, disciplinary backgrounds, years of experience, and other important intersectional factors, thus limiting the analysis. Another limitation is that this research did not analyze existing monitoring and evaluation (M&E) data. Notably, the perspectives documented in this research require further testing and M&E to ensure the practices are suitable and useful in different communities.

## Conclusion and future directions

This study found social work in complex emergencies not only matters, but also it can be lifesaving by supporting children to stay in school and preventing suicide. However, it requires careful consideration to ensure social work is *reactivating* rather than *replacing* traditionally effective forms of support that lie within the social fabric of families and communities. As institutions are strained in complex emergencies, and there can be difficulties with accessing support, social work practice must incorporate locally supported family and community roles. This study found the protracted nature of the Syrian crises generated a need for social work practice to re-establish Syrian families and communities’ supportive roles. Practitioners viewed “good” social work practices as those which helped reactivate the family and community's supportive roles in a manner suitable to the community. Conducting “good” social work practice requires careful consideration of who is practicing social work and how such practices are aligned with the culture, especially in a setting such as NWS that did not have a formal social work profession before the crisis. This research documents insights on social work practice and theory in NWS that could contribute to future social work development and validates the sophisticated and thoughtful practices Syrian practitioners are doing to benefit their communities.

Future research could analyze relevant M&E data to triangulate the findings, and continue to include the perspectives of families and communities who have accessed support from practitioners who perform social work functions in NWS or other complex emergencies. Future scholarship might also explore perspectives of effective social work practice in other complex emergencies; “good” practices with specific populations; and compare perspectives on “good” practices between contexts. Studies can also continue to analyze the broader historical and societal factors influencing social work in NWS. Since Syrian practitioners are part of the community, future research should explore if Syrian practitioners’ experience tension between promoting the family and communities’ natural forms of healing *and* needing to perform the functions families and communities performed before the crisis.

Despite the challenges experienced by social workers in conflict and ongoing upheaval, creative and innovative social work practices emerge (Harrop & Ioakimidis, 2018). This research suggests the importance of considering how social work practice can help to reactivate the family and community's supportive role, particularly in a context where social work is a new role necessary to address needs arising from the complex emergency in a manner suitable to the community. The practitioners who have documented their learnings, and dilemmas through this research provide a source of hope and strength for social work in complex emergencies. As one Syrian practitioner expressed: “there's an adage that's like this: ‘what harms maybe can be beneficial’ … inshallah that we with our experience that we went through with our lives be beneficial to others of this humanity that we all belong to.” As the social work profession plays a key role in complex emergencies, social work researchers must continue to consider how to conduct research that ultimately supports those who perform social work functions during complex emergencies.
